# Self-Adaptable Tropos Catalysts

**DOI:** 10.1021/acs.accounts.1c00326

**Published:** 2021-08-04

**Authors:** Montserrat Diéguez, Oscar Pàmies, Christina Moberg

**Affiliations:** † Departament de Química Física i Inorgànica, Universitat Rovira i Virgili, C/Marcel·lí Domingo 1, 43007 Tarragona, Spain; ‡ Organic Chemistry, Department of Chemistry, KTH Royal Institute of Technology, SE 10044 Stockholm, Sweden

## Abstract

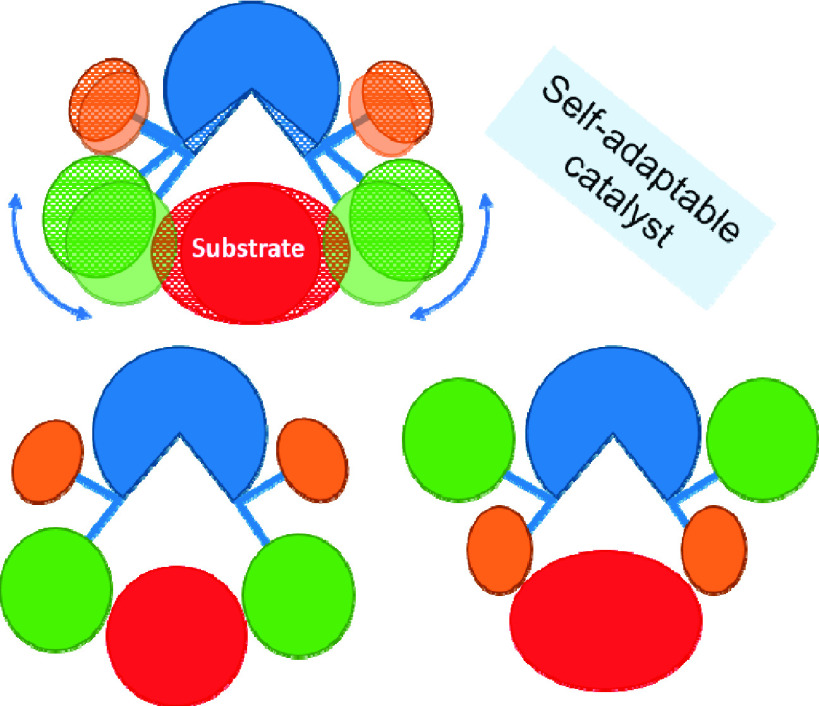

Biological systems have often served as inspiration for the design of synthetic catalysts. The lock and key analogy put forward by Emil Fischer in 1894 to explain the high substrate specificity of enzymes has been used as a general guiding principle aimed at enhancing the selectivity of chemical processes by optimizing attractive and repulsive interactions in molecular recognition events. However, although a perfect fit of a substrate to a catalytic site may enhance the selectivity of a specific catalytic reaction, it inevitably leads to a narrow substrate scope, excluding substrates with different sizes and shapes from efficient binding. An ideal catalyst should instead be able to accommodate a wide range of substrates—it has indeed been recognized that enzymes also are often highly promiscuous as a result of their ability to change their conformation and shape in response to a substrate—and preferentially be useful in various types of processes. In biological adaptation, the process by which species become fitted to new environments is crucial for their ability to cope with changing environmental conditions. With this in mind, we have been exploring catalytic systems that can adapt their size and shape to the environment with the goal of developing synthetic catalysts with wide scope.

In this Account, we describe our studies aimed at elucidating how metal catalysts with flexible structural units adapt their binding pockets to the reacting substrate. Throughout our studies, ligands equipped with tropos biaryl units have been explored, and the palladium-catalyzed allylic alkylation reaction has been used as a suitable probe to study the adaptability of the catalytic systems. The conformations of catalytically active metal complexes under different conditions have been studied by both experimental and theoretical methods. By the design of ligands incorporating two flexible units, the symmetry properties of metal complexes could be used to facilitate conformational analysis and thereby provide valuable insight into the structures of complexes involved in the catalytic cycle. The importance of flexibility was convincingly demonstrated when a phosphine group in a privileged ligand that is well-known for its versatility in a number of processes was exchanged for a tropos biaryl phosphite unit: the result was a truly self-adaptive ligand with dramatically increased scope.

## Key References

- ZalubovskisR.; BouetA.; FjellanderE.; ConstantS.; LinderD.; FischerA.; LacourJ.; PrivalovT.; MobergC.Self-Adaptable Catalysts: Substrate-Dependent Ligand Configuration. J. Am. Chem. Soc.2008, 130, 1845–18551819886710.1021/ja074044k.^1^*Flexible ligands were shown to adapt their configuration to intermediates involved in Pd-catalyzed allylic alkylations.*
- MazuelaJ.; NorrbyP.-O.; AnderssonP. G.; PàmiesO.; DiéguezM.Pyranoside Phosphite–Oxazoline Ligands for the Highly Versatile and Enantioselective Ir-Catalyzed Hydrogenation of Minimally Functionalized Olefins. A Combined Theoretical and Experimental Study. J. Am. Chem. Soc.2011, 133, 13634–136452176187210.1021/ja204948k.^2^*The flexibility of the biphenyl phosphite group was the key to achieving high enantioselectivities in the hydrogenation of a wide range of olefins with different substitution patterns and functional groups.*
- BelliniR.; MagreM.; BioscaM.; NorrbyP.-O.; PàmiesO.; DiéguezM.; MobergC.Conformational Preferences of a Tropos Biphenyl Phosphinooxazoline–A Ligand with Wide Substrate Scope. ACS Catal.2016, 6, 1701–1712.^3^*The unusually wide substrate scope of the ligand was shown to be a result of its ability to change the size of the substrate-binding pocket.*

## Introduction

Enantioselective transition metal catalysis is an enabling technology for the preparation of enantiomerically enriched chiral compounds, and extensive efforts during the last decades have resulted in metal catalysts with impressive performances that are capable of efficient chirality transfer in a variety of synthetically significant processes.^4^

The preparation of ligands is usually the bottleneck in catalyst optimization. To date, efficient ligands have commonly been identified using empirical methods, ranging from trial-and-error approaches to more or less rational design.^5^ For the efficient evaluation of catalyst performance, a variety of procedures have been developed, including high-throughput screening technologies, today increasingly by the use of high-efficiency robotics.^6^

Such empirical methods are supplemented or even being replaced by computationally guided methods. Today calculations permit accurate estimations of differences in transition state energies and thus have the ability to predict reaction outcomes. In recent years artificial intelligence and machine learning^7^ have become increasingly explored tools for making predictions of quantitative structure–reactivity and structure–selectivity relationships^8^ as well as predictions of activation energies.^9^

Although careful ligand design can lead to highly selective catalytic systems, such systems are usually limited to specific reactions and often even to particular types of substrates. In order to reduce time-consuming ligand synthesis and fine-tuning of catalyst structures, catalysts with wide reaction applicability and substrate scope are desirable. To satisfy this need, a series of so-called privileged ligands have been identified.^10^ An attractive alternative to those ligands classified as privileged consists of ligands with stereochemical flexibility.^11^ The authors of this Account are particularly interested in flexible ligands that are capable of adapting their structure to a reacting substrate.

## Background

**Figure 1:**
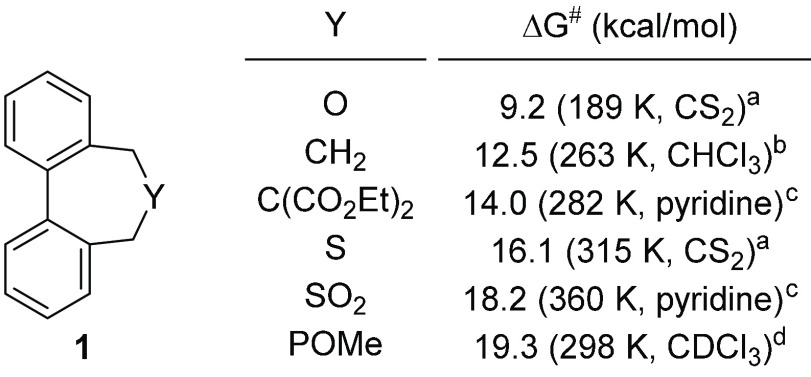
Barriers for tropoisomerization of bridged biaryl derivatives. Data were taken from (a) ref (14), (b) ref (15), (c) ref (16), or (d) ref (17).

Conformationally flexible *tropos* ligands with the ability to switch between two states have been successfully applied in a variety of catalytic processes.^12^ Among available flexible ligands, biphenyl derivatives have most commonly been employed. Biphenyls bridged by three-atom units at the 2- and 2′-positions (**1**) have nonplanar structures and thus are chiral. The conformational mobilities of such biphenyls have been subjected to extensive studies, and energy barriers for conformational change for a range of derivatives have been determined, most commonly by NMR spectroscopy or theoretical calculations. The barriers are largely dependent on the structure of the bridging unit (Figure 1). A half-life of at least 1000 s at a given temperature has been regarded as a requirement for the isomers to be separable.^13^

**Figure 2:**
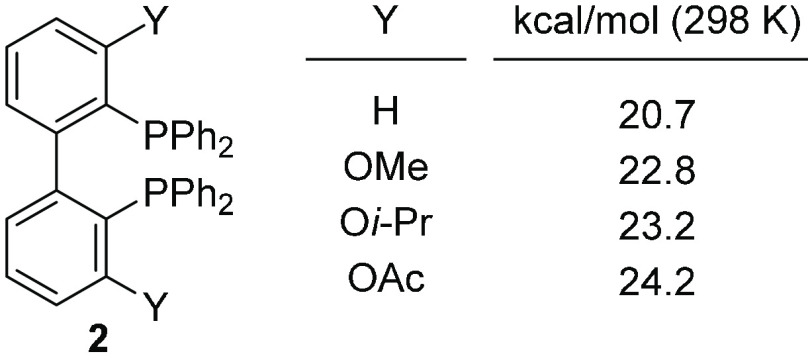
Inversion barriers determined by dynamic high-performance liquid chromatography. Data were taken from ref (19).

Metal complexes of 2,2′-bis(diphenylphosphino)-1,1′-biphenyl (BIPHEP, **2**, Y = H) have been extensively used in catalytic applications. The barrier to tropoinversion in the parent ligand was determined by dynamic NMR spectroscopy to be 22 ± 1 kcal/mol (398 K)^18^ and later by enantioselective dynamic high-performance liquid chromatography to be 20.7 kcal/mol (298 K).^19^ The barriers for inversion of 3,3′-disubstituted derivatives are somewhat higher (Figure 2), whereas substituents at the 5- and 5′-positions have less influence on the barrier.^19^

**Figure 3:**
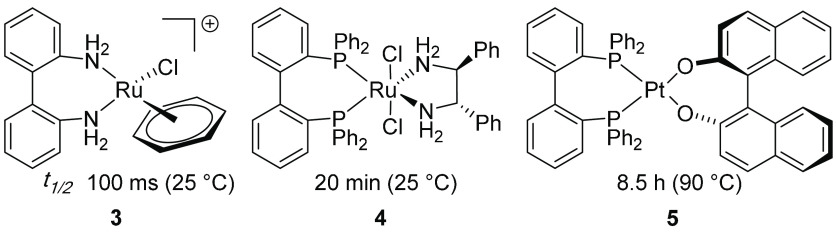
Rates of tropoisomerization for biphenyldiamine and BIPHEP complexes.

In metal complexes with BIPHEP, as well as with analogous nitrogen ligands, the rates of configurational change differ largely depending on the metal and the structure of the ligands, as illustrated by complexes **3**, **4**, and **5**, with half-lives (*t*_1/2_) of ∼100 ms at 25 °C, 20 min at 25 °C, and ∼8.5 h at 90 °C,^20^ respectively (Figure 3).

**Figure 4:**
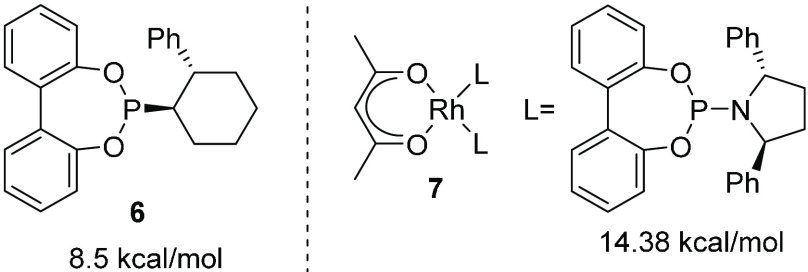
Inversion barriers for a biphenyl-based phosphite ligand and a metal complex.

The barriers to conformational change in biphenyl-based phosphite derivatives (Figure 4) are considerably lower than those in tropos biphenyls with bridging C instead of O, with that of **6** being 8.5 kcal/mol (197 K).^21^ As expected, the barriers in metal complexes are higher, although considerably lower than those in complexes with BIPHEP, as illustrated by a barrier of 14.38 kcal/mol for **7** (320 K, toluene-*d*_8_; Figure 4).

### Configurational Control

**Figure 5:**
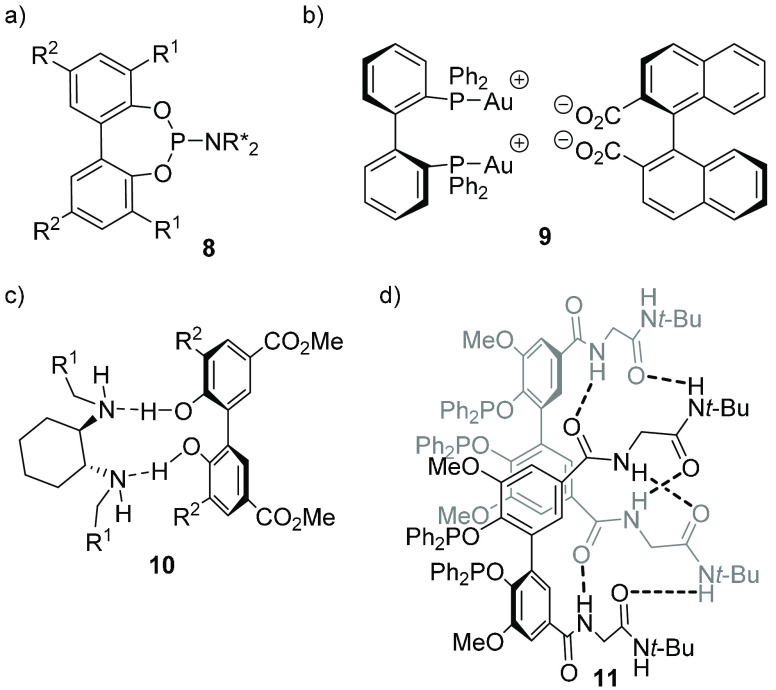
Conformational control by (a) a chiral group attached to the ligand, (b) a chiral counterion, (c) supramolecular hydrogen bonding, and (d) other supramolecular interactions.

The configuration of the tropos unit can more or less efficiently be controlled by a chiral group attached to the flexible unit (as in ligand **8**; Figure 5a)^22^ as well as by a separate chiral ligand bound to the same metal center as the biaryl ligand; a diastereomeric ratio as high as 100:0 was observed for the *N*,*N*-dimethyl analogue of Ru complex **4** (Figure 3).^23^ Chirality control can also be achieved by virtue of a chiral anion, as demonstrated for BIPHEP Au complex **9** (Figure 5b),^24^ as well as by supramolecular hydrogen-bonding interactions (adduct **10**; Figure 5c)^25^ and other supramolecular interactions;^26^ through the formation of supramolecular dimers, essentially complete control of the axial chirality of biaryls equipped with amino acid-derived substituents at the 5- and 5′-positions was achieved (compound **11**; Figure 5d).^27^

### Mechanism of Tropoinversion

**Scheme 1:**
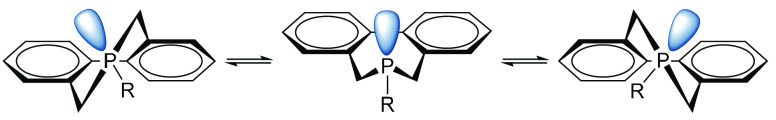
Tropoinversion of Phosphenines

Configurational switching in biphenyl derivatives proceeds via a planar transition state, and the barrier to tropoisomerization thus depends on the ease with which the biphenyl system can pass through a planar conformation.^28^ In phosphenines, pyramidal inversion at phosphorus is required, but since P is not a stereogenic center, this does not affect the chirality (Scheme 1).^17^

In metal complexes, tropoisomerization may occur “on-metal”, without decoordination of a ligand arm, but one-arm decoordination may also be required for configurational change to occur.

**Scheme 2:**
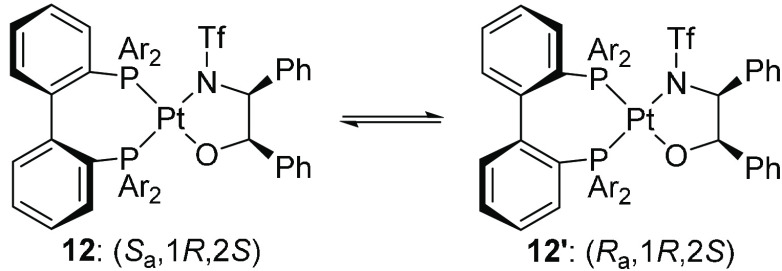
Tropoisomerization in a Square-Planar d^8^ Pt(II) Complex Requires Excess Ligand

Square-planar d^8^ Pt(II) complexes **12** prefer to react via associative mechanisms, making decoordination of one ligand arm unfavorable. A ca. 1:1 mixture of the diastereomeric pairs of ligands ±**12** and ±**12**′ (Scheme 2) was stable toward isomerization but underwent isomerization in the presence of excess ligand, suggesting a mechanism involving ligand–ligand exchange and no tropoinversion.^20^ Analogous results were obtained in reactions where the N,O-ligand was replaced by BINOL. Isomerization in the absence of ligand–ligand exchange required elevated temperatures, with *t*_1/2_ = 8.5 h at 90 °C for the BINOL complexes (Figure 3). As expected for an arm-off mechanism, the isomerization was accelerated by donor ligands such as pyridine, but whether the isomerization in the absence of a donor ligand proceeded via a planar seven-membered metallacycle or a one-arm-off mechanism was unclear.

Tropoinversion in the octahedral Ru complex **4** (Figure 3) (*t*_1/2_ ≈ 20 min at 25 °C) was found to involve decoordination and solvent-assisted rotation around the biphenyl single bond and subsequent recoordination of phosphine to the Ru center,^29^ whereas tropoinversion in **3** (*t*_1/2_ ≈ 100 ms; Figure 3) as well as in the analogous Os complex did not involve isomerization at the metal center and thus not cleavage of a nitrogen–metal bond.^30^

**Figure 6:**
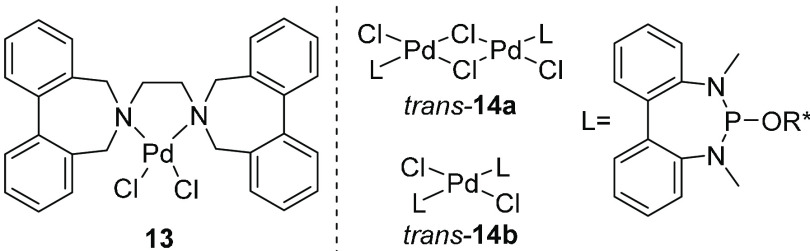
Examples of bridged and mononuclear complexes with tropoisomerization proceeding without decoordination of the ligand.

In complexes where the metal resides outside the bridge connecting the phenyl rings, as in **13**,^31^ and in bridged and mononuclear Pd complexes **14** (with, e.g., R* = (−)-menthyl),^32^ isomerization proceeds while the ligand is coordinated to the metal (Figure 6). The barrier to tropoisomerization in **13** was found to be 10.6 ± 0.8 kcal/mol at 258 K (11.1 ± 1.6 kcal/mol for the free ligand), and those in the di- and mononuclear complexes *trans*-**14a** and *trans*-**14b** were 15.4 ± 0.1 and 15.7 ± 0.2 kcal/mol, respectively, at 298 K (12.7 ± 0.6 kcal/mol in the free ligand).

## Catalytic Applications

**Scheme 3:**
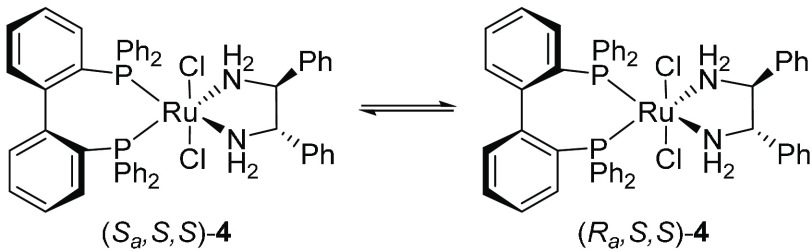
Tropoisomerization in Ru(II) complex

Our groups have had a long-standing interest in the use of metal complexes with flexible ligands in asymmetric catalysis, prompted by their ability to exert configurational control. Prior to our work, a number of successful studies had been reported, starting with the use of flexible Rh(I) complexes for catalytic hydroformylation^33^ and flexible Ti(IV) complexes of 2,2′-dihydroxybiphenyl (BIPOL) in combination with enantiopure diols as enantioselective Lewis acid catalysts.^34^ Mikami and Noyori used Ru complexes of BIPHEP in asymmetric catalysis and were able to control the configuration by virtue of a second coordinating chiral ligand.^35^ Activation of a Ru(II) chloride complex by (*S*,*S*)-1,2-diphenylethylenediamine (complex **4**; Figure 3) initially afforded an equimolar mixture of diastereomers. Upon standing in chloroform/isopropanol, a 3:1 mixture of the (*S*_*a*_,*S*,*S*) and (*R*_*a*_,*S*,*S*) diastereomers was formed (Scheme 3). Higher enantioselectivities than anticipated were observed in the catalytic hydrogenation of prochiral ketones as a consequence of the higher efficiency of the major diastereomer. With more substituted BIPHEP derivatives, complete epimerization was observed,^23^ and enantiomerically pure complexes could be isolated via enantiomer-selective complexation of a chiral enantiopure amine to racemic BIPHEP–Ru complexes.^36^ The topic has been extensively explored by Mikami and coworkers.^28^

**Figure 7:**
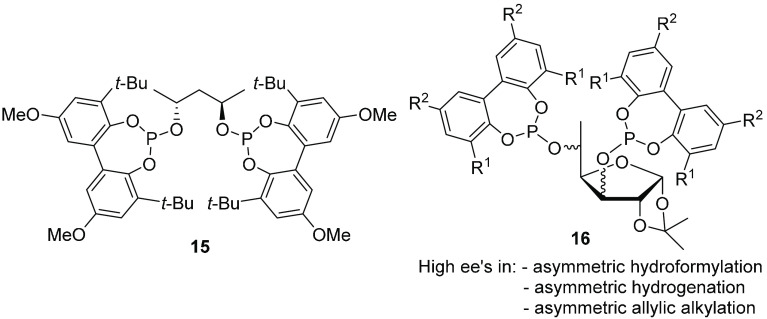
Examples of biphenyl diphosphite ligands successfully applied in asymmetric catalysis.

Atropisomerism induced by a chiral motif incorporated into the flexible ligand structure instead of in a separate ligand has also been explored in catalytic reactions. Two of the present authors have reported a large number of examples based on biphenolphosphites.^37^ The use of *C*_2_-symmetric diphosphite ligand **15** had early been shown to display high enantioselectivities in the Rh-catalyzed hydroformylation of styrenes (Figure 7), with ee’s comparable to those observed using the corresponding rigid binaphthyl ligand.^33^ The key to success was found to be the introduction of substituents at the 3- and 3′-positions, as this helped to control the tropoisomerization. This finding inspired us to design other bulky biphenyl phosphite ligands for use in catalysis (see, e.g., ligands **16**).^37^

**Scheme 4:**
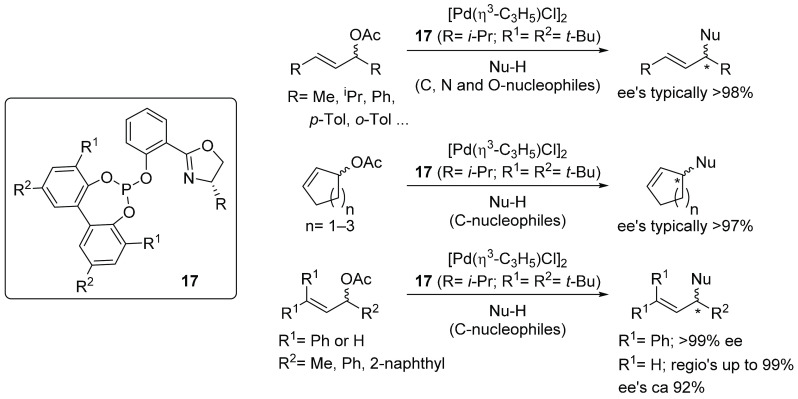
Summary of the Pd-Catalyzed Allylic Substitution Reactions Using Ligand **17**

The efficiency of biphenyl-based ligands also extends to heterodonor bidentate ligands. One of our groups introduced a phosphine–oxazoline (PHOX) analogue with a flexible phosphite unit and were able to overcome the high substrate specificity in the Pd-catalyzed allylic substitution reaction (vide infra).^38^ With ligands **17** (R = Ph, *i*-Pr; R^1^ = R^2^ = *t*-Bu), which contain a bulky biphenyl phosphite moiety, excellent enantioselectivities were reached in the allylic substitution of a wide number of substrates and C-, N-, and O-nucleophiles (up to 60 compounds in total).^3^ Enantioselectivities of up to 99% ee were reached in the allylic substitution of linear symmetrical 1,3-disubstituted substrates with different steric and electronic properties, cyclic substrates with different ring sizes, and challenging nonsymmetrically mono- and trisubstituted substrates (Scheme 4).^3,38^

**Figure 8:**
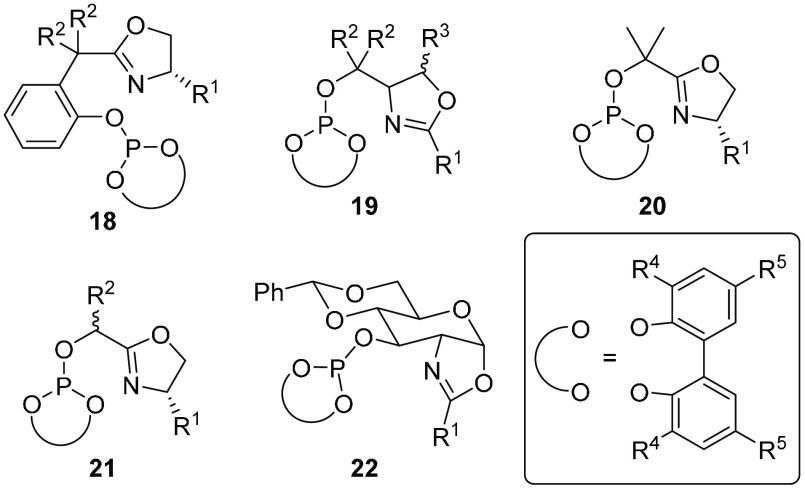
Flexible biphenyl phosphite–oxazoline ligands.

Interestingly, ligand **17** was also successfully applied in other asymmetric reactions such as hydrogenations,^39^ hydroborations,^40^ and intermolecular Heck reactions.^41^ By changing the backbone of ligands **17** (ligands **18**–**22**; Figure 8), the substrate scope was even further improved, with results comparable to or surpassing those for the best catalysts reported to date.^42^ Among the ligands with different backbones, we can highlight pyranoside phosphite–oxazoline ligand **22**, which allowed efficient applications in allylic substitutions,^43^ Heck reactions,^44^ and hydrogenations.^2^ For example, in the hydrogenation of challenging unfunctionalized olefins, excellent enantioselectivities (up to 99% ee) were achieved with many tri- and 1,1′-disubstituted olefins, even with the highly challenging *Z* isomers, triarylsubstituted substrates, and olefins with relatively poorly coordinating groups, such as α,β-unsaturated esters and ketones, vinylsilanes, allylic alcohols, and acetates as well as vinylboronates (44 examples in total).^2^ DFT studies in collaboration with P.-O. Norrby confirmed that the flexibility of the biaryl phosphite group is crucial for achieving high enantioselectivities.^2^ Thus, even though according to the optimized DFT structures of the transition states the biphenyl group adopts the *R* configuration in complexes with both *E*- and *Z*-olefins, the flexibility of the tropos unit confers to the Ir catalysts the ability to adapt its chiral pocket to the substrate, leading to high enantioselectivity in reactions with both types of olefins using the same ligand **22** (R^1^ = Ph; R^4^ = R^5^ = *t*-Bu). Analogous behavior was observed for ligand **17** in the Pd-catalyzed allylic substitution discussed later.

## Configuration Induced by an External Agent

**Scheme 5:**
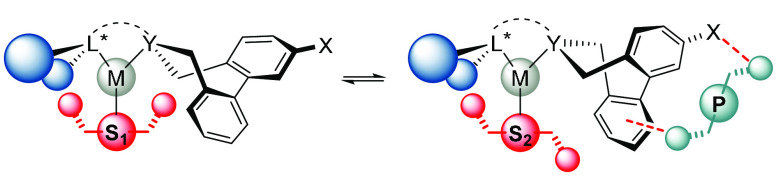
Influence of Different Substrates (S_1_ and S_2_) and a Product (P) on the Configuration of the Tropoisomeric Biaryl Unit

The ability of a chiral element, either covalently attached to the flexible unit or present in a separate ligand coordinated to the catalytic center, to control the stereochemistry of a chirally flexible biaryl function results either in a single diastereomer or more often in a mixture of two diastereomers, usually with different reactivities. The position of the equilibrium may be influenced by the reacting substrate, with different substrates possibly favoring different configurations of the ligand, as well as by the product formed in the reaction (Scheme 5).

### Product-Induced Conformations

**Scheme 6:**
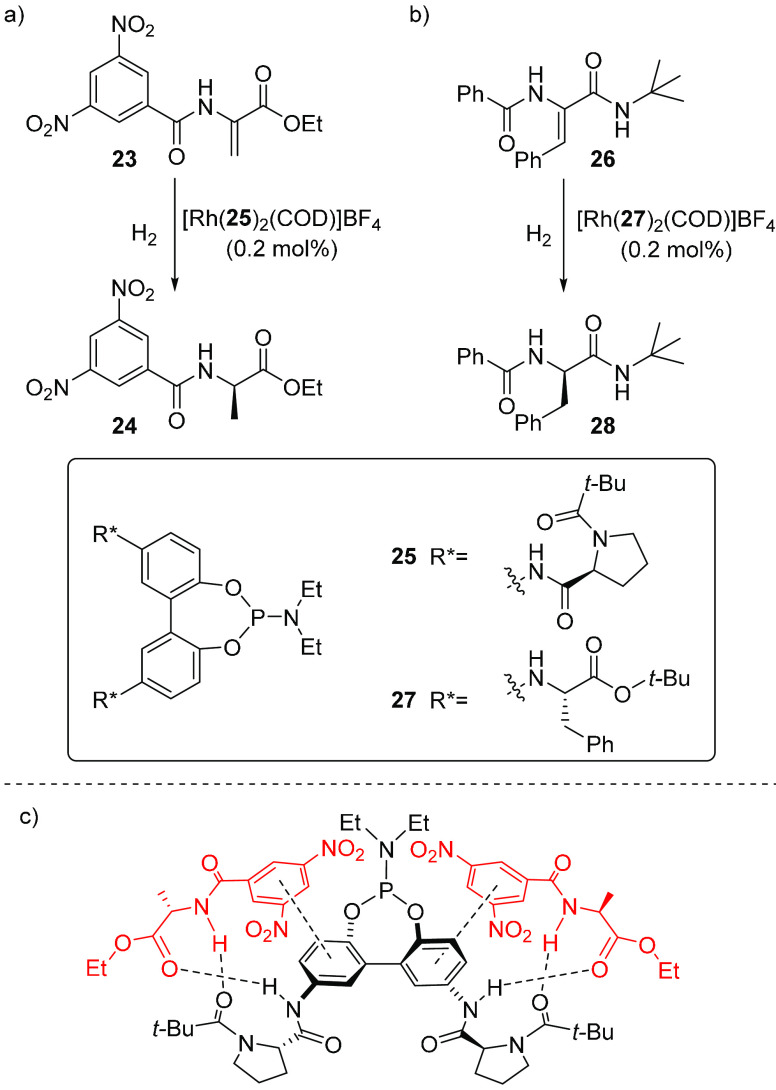
(a, b) Product-Induced Conformations in Asymmetric Hydrogenation of (a) **23** Using [Rh(**25**)_2_(COD)]BF_4_ and (b) **26** Using [Rh(**27**)_2_(COD)]BF_4_; (c) Non-covalent Interactions between the Product and the Ligand

As shown by Trapp and co-workers, selective interaction of one product enantiomer from a (moderately) enantioselective reaction with a tropos catalyst may lead to autoinduction and thus increasing enantioselectivity with time. This was demonstrated in the catalytic hydrogenation of amino acid precursor **23** (Scheme 6a). Supramolecular interaction of **24**, the chiral product from hydrogenation of **23** catalyzed by the Rh complex containing tropoisomeric biphenylphosphoramidite **25** equipped with recognition sites derived from amino acids at the 5- and 5′-positions, led to self-amplification of the chirality via interaction of one product enantiomer with the chiral selector (Scheme 6c).^45^ The ee of the product obtained in the absence of interacting product was estimated as 63%, with an excess of the *R* enantiomer. In contrast, the use of only 0.2 mol % catalyst led to the opposite enantiomer with 71% ee as a result of the preferred interaction of one of the product enantiomers.

In another example, a stoichiometric reaction gave the product from hydrogenation of **26** with 19% ee in favor of the *S* enantiomer using an analogous Rh complex equipped with a different chiral selector (ligand **27**).^46^ With a lower catalyst loading, the initially preferred product enantiomer interacted with the catalyst, which caused a switch of the selectivity, resulting in the *R* product **28** with 41% ee (Scheme 6b).

### Substrate-Induced Conformations

The ability of a substrate to influence the configuration of the tropoisomeric moiety may result in a catalyst that can tolerate a wide range of substrates, thereby limiting the need for ligand preparation. We have used Pd-catalyzed allylic alkylation^47^ as a suitable model process for the assessment of the ability of a ligand to respond to the substrate. Most catalysts employed for this process show a pronounced substrate specificity, with linear, sterically bulky (“broad”) substrates requiring different ligands than small, unhindered (“narrow”) substrates.^47^

**Figure 9:**
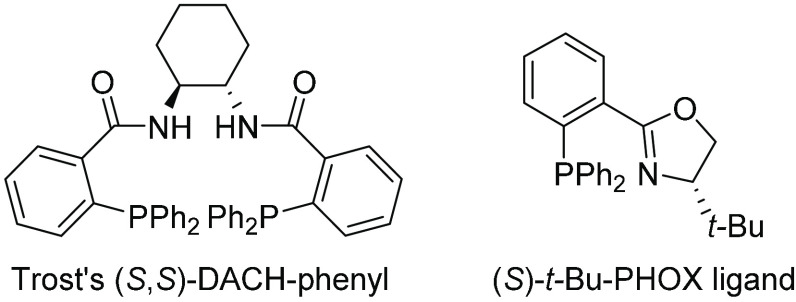
Representative Trost (*S*,*S*)-DACH-phenyl diphosphine and phosphine–oxazoline (*S*)-*t*-Bu-PHOX ligands.

Two of the most versatile types of ligands are the PHOX family of ligands^48^ and the Trost diphosphine type of ligands (Figure 9).^49^ PHOX ligands, which interact with the substrate mainly at its wings, provide high enantioselectivities for “broad” substrates, while the Trost ligand, equipped with a small pocket, gives products with high enantiomeric ratios primarily from “narrow” substrates. Allylic alkylation therefore serves as a suitable benchmark reaction to assess the adaptability of a catalyst.

**Scheme 7:**
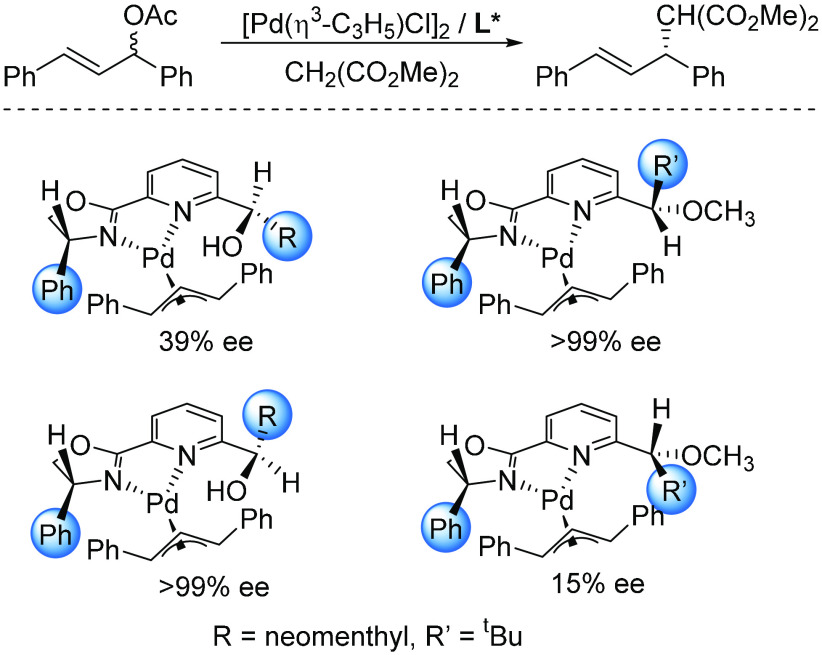
Representative Results on the Use of Hydroxymethyl-Substituted Pyridyloxazolines and Their Methylated Counterparts in Pd-Catalyzed Asymmetric Allylic Substitution

Early results from one of our laboratories inspired studies of catalysts capable of conformational change. In Pd(0) η^2^-olefin complexes, hydroxymethyl-substituted pyridyloxazolines adopt conformations different from those of the methylated analogues as a result of a stabilizing OH–Pd(0) interaction.^50,51^ In reactions of malonate with *rac*-1,3-diphenylpropenyl acetate, a “broad” substrate, the two types of ligands led to largely different results (Scheme 7), thus demonstrating the crucial relation between the conformation of the ligand and the size of the substrate.^52^

**Scheme 8:**
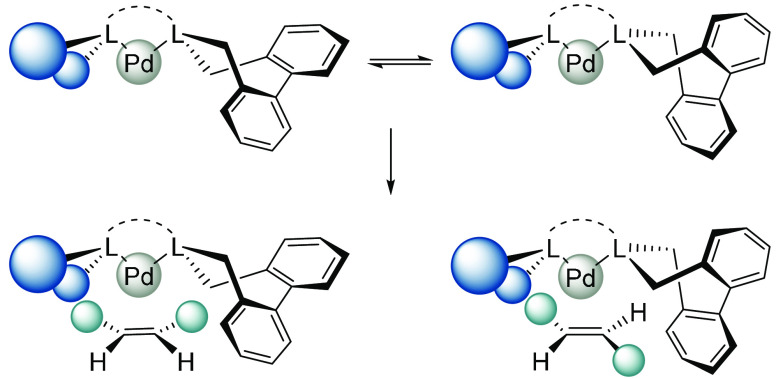
Biaryl Configuration Adapted to the Coordinating Olefin

However, to be synthetically useful, ligands that did not require chemical modification for conformational change were needed. Ligands equipped with a conformationally flexible motif capable of shifting between two states and a rigid chiral element were assumed to fulfill this requirement, since the flexibility offered by a biphenyl moiety can be used to fine-tune the chiral pocket formed upon complexation. Phosphepine and azepine ligands were selected as suitable structures for the studies (Scheme 8).

**Figure 10:**
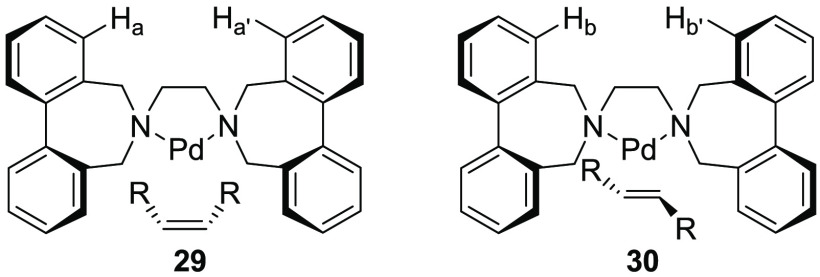
*Z*-Olefin coordinated to a complex with the ligand in the *C*_*s*_ conformation and *E*-olefin coordinated to a complex with the ligand in the *C*_2_ conformation. In each case, one out of two complexes is shown.

The ability of a substrate to affect the configuration of biaryl units was demonstrated by studies of Pd(II) allyl and Pd(0) olefin complexes with 1,2-bis[4,5-dihydro-3*H*-dibenzo[c–e]azepino]ethane (Figure 10).^1^ Complexes with symmetrically substituted substrates (1,3-disubstituted *syn*,*syn*- and *anti*,*anti*-η^3^-allyl groups and 1,2-disubstituted *Z*-olefins) and the ligand in the (*R*_a_*,*S*_a_*) conformation have *C*_*s*_ symmetry and pairwise-enantiotopic protons (e.g., H_a_ and H_a′_ in **29**), whereas in a complex of an *E*-olefin and the ligand in the same conformation, all protons would be nonequivalent. In contrast, the (*R*_a_*,*R*_a_*) conformation would lead to a *Z*-olefin complex with *C*_1_ symmetry and all protons different but a *C*_2_-symmetric *E*-olefin complex with pairwise-homotopic protons (e.g., H_b_ and H_b′_ in **30**). The conformation of the ligand could thus be determined using symmetry arguments.

**Figure 11:**
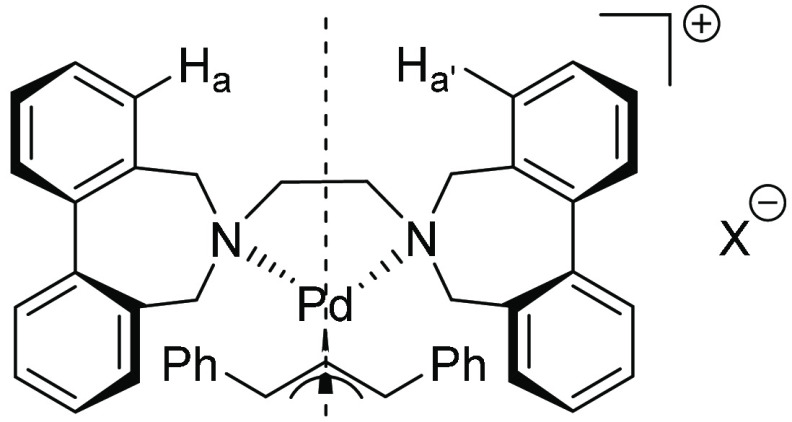
H_a_ and H_a′_ are enantiotopic when X is achiral but diastereotopic when X is chiral.

The η^3^-cyclohexenyl Pd(II) complex with the “bis-flexible” ligand was obtained as a single isomer, while a major isomer of the η^3^-1,3-diphenylpropenyl complex was formed along with a 3% yield of a minor isomer. In both cases, the *C*_*s*_ configuration of the ligand was preferred, as elucidated by comparison of the NMR spectra of the complexes with those of the analogous rigid bis(binaphthyl) complexes and corroborated by DFT calculations.^1^ This conclusion was further supported by the observation that enantiotopic became diastereotopic by replacement of PF_6_^–^ with a chiral counteranion (Figure 11).

**Figure 12:**
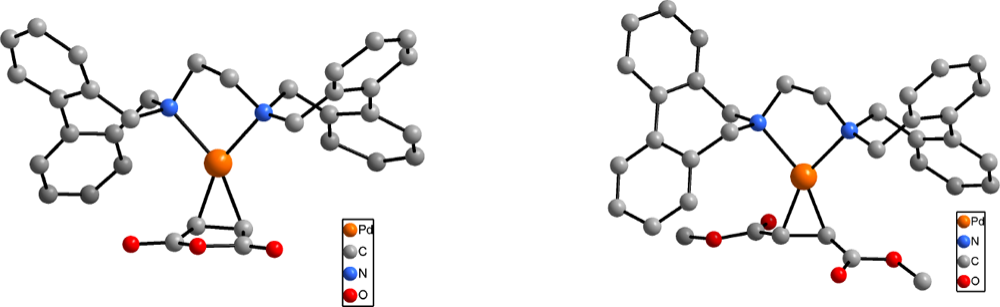
X-ray structures of Pd(0) complexes **29** (left) and **30** (right) with maleic anhydride and dimethyl fumarate as *Z*- and *E*-olefins, respectively. Reproduced from ref (1). Copyright 2008 American Chemical Society.

To simplify the synthesis and analysis of the Pd(0) olefin complexes, maleic anhydride and dimethyl fumarate were used as model olefins to mimic the product olefin complexes obtained from attack by the nucleophile. Single complexes were obtained from both olefins: in complexes with *Z*- and *E*-olefins, the ligand was found to adopt conformations with *C*_*s*_ and *C*_2_ symmetry, respectively. The configurations of the ligand in the two types of complexes were unambiguously confirmed by X-ray crystallography (Figure 12).

**Figure 13:**
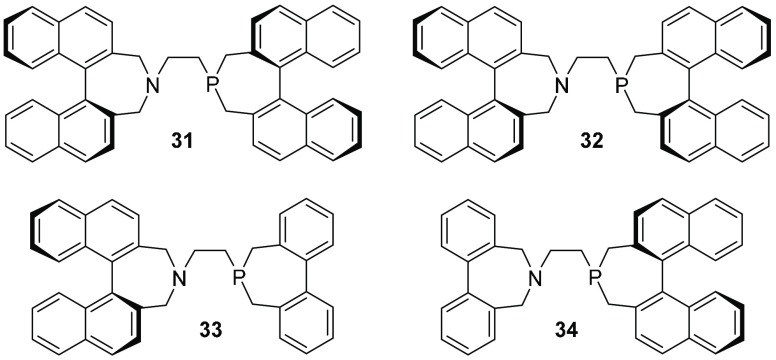
Rigid and “semiflexible” phosphepine–azepine ligands.

These studies demonstrate that in the Pd(0) complexes, the flexible ligand adapts its structure to the coordinated olefin. Although catalytic reactions using the ligands with two flexible units evidently can provide only racemic products, truly chiral analogues were used to assess the structural preferences for different types of substrates. Studies of rigid phosphepine and azepine ligands showed that sterically hindered, linear substrates prefer ligands with *C*_2_ or pseudo-*C*_2_ symmetry (e.g., **31**; Figure 13), whereas smaller substrates provide higher selectivities when ligands with pseudo-*C*_*s*_ symmetry (e.g., **32**) are used.^53^

Analogues with one flexible biphenyl element and a fixed element of chirality (e.g., **33** and **34**) can adopt pseudo-*C*_s_ as well as pseudo-*C*_2_ symmetry and were thus expected, depending on the substrate, to behave as either type of ligand (**31** or **32**) in the catalytic reactions.

**Scheme 9:**
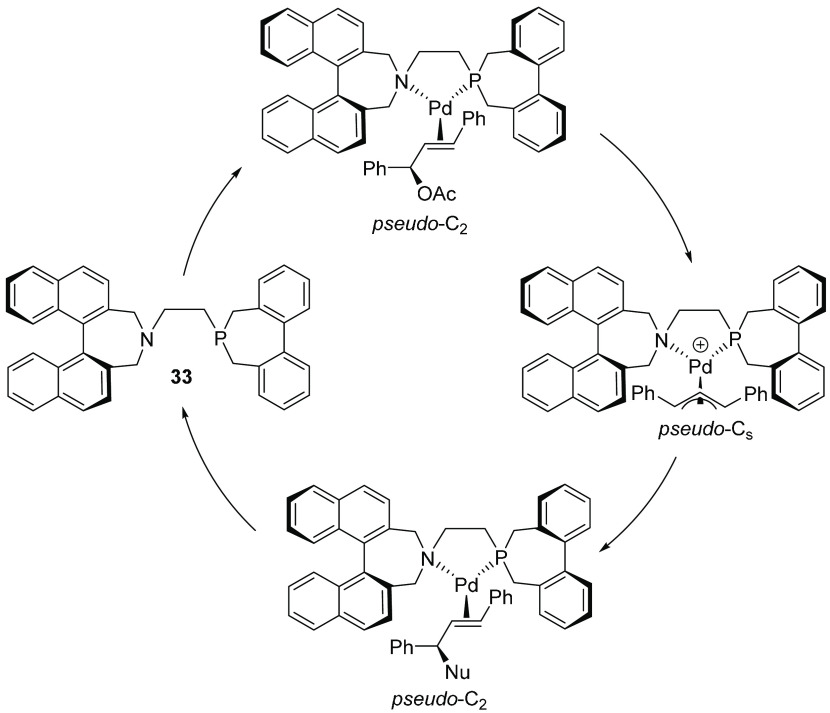
Mechanism of Pd-Catalyzed Allylic Alkylation Illustrating Tropoinversions Occurring along the Catalytic Cycle

As expected, poor selectivity was observed in the reaction of *rac*-1,3-diphenylpropenyl acetate using pseudo-*C*_*s*_-symmetric ligand **32** (37% ee and low reactivity). However, although excellent enantioselectivity was observed in the same reaction using pseudo-*C*_2_-symmetric ligand **31** and the analogous N,N-ligand (98 and 99% ee, respectively), lower selectivity was observed in reactions with the corresponding flexible ligands **33** and **34** (81 and 87% ee). The lower selectivity is probably due to required tropoisomerization of the ligand during each step along the catalytic cycle (Scheme 9) combined with the relatively high barrier, causing the ligand to behave as a 1:1 mixture of the *C*_*s*_- and *C*_2_-symmetric ligands. Quite poor selectivities, although somewhat higher for the *C*_s_-symmetric ligand **32** than for **31**, were observed in reactions with cyclic “narrow” substrates. These results are in line with the assumption of a late transition state and provide an explanation for the different behaviors of the two types of ligands.

**Figure 14:**
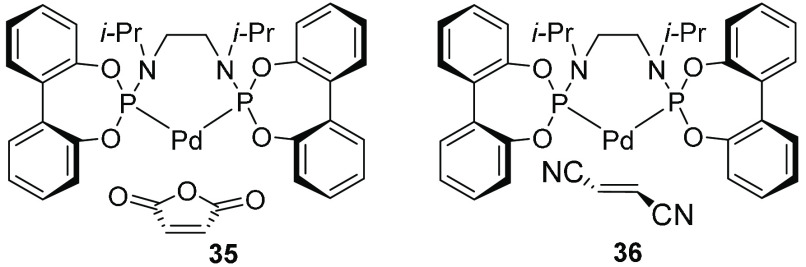
*C*_*s*_-symmetric and *C*_2_-symmetric Pd(0) complexes with *Z*- and *E*-olefins, respectively.

Although the ability of flexible ligands to adapt to the coordinating substrate/product was demonstrated, a flexible unit with a lower barrier to conformational change was needed to achieve an efficient self-adaptable catalytic system. Phosphites and phosphoramidites are suitable candidates for ligands capable of adapting their conformation analogously, as shown for example by single ^31^P NMR resonances from Pd(0) complexes **35** and **36** with *Z*- and *E*-olefins, respectively (Figure 14).^54^

**Figure 15:**
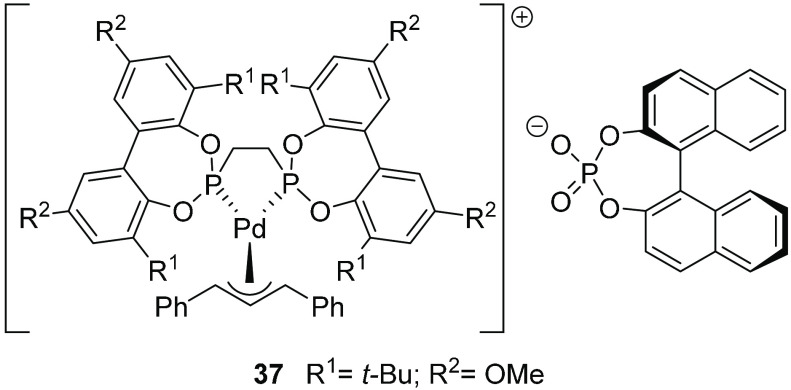
Cationic Pd–allyl complex **37** with a flexible bisphosphonite ligand and a chiral counteranion.

The ability of a chiral counteranion to differentiate enantiotopic groups in Pd(II) η^3^-allyl complexes with flexible bisazepine ligands (Figure 11)^1^ motivated attempts to use flexible bisphosphonites in the presence of a chiral counteranion (Figure 15). However, only racemic product was observed. This is in fact not surprising, since the transition state is expected to resemble the product neutral olefin complex rather than the ionic η^3^-allyl complex, and the counterion is therefore expected to exert only a minor influence on the stereochemistry-controlling step of the catalytic reaction.

However, if the chiral anion could be forced to be permanently attached to the catalyst also in an olefin complex with a flexible ligand, an influence on the stereochemical outcome of the reaction may be expected. Dydio, Reek, and co-workers had developed systems fulfilling this requirement, so-called cofactor ligands, where a readily available chiral anion, a cofactor, is bound to a cationic pocket in the ligand.^55^

**Figure 16:**
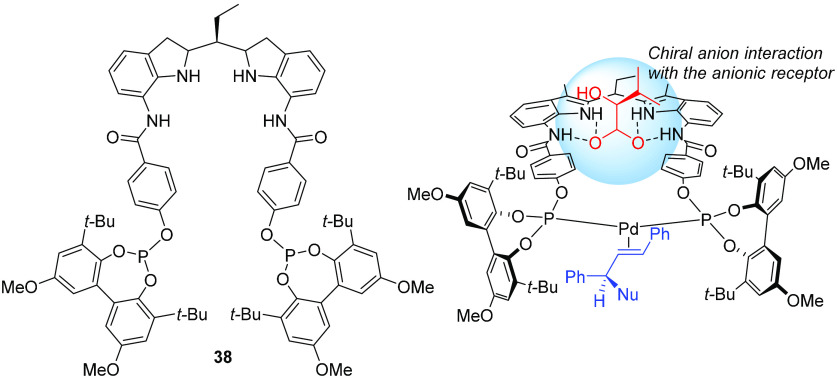
Flexible diphosphite ligand **38** and model for the (*S*)-2-hydroxy-3-methylbutyrate-controlled chiral transfer to the tropos units.

In collaboration with the Reek group, a tropos ligand (Figure 16) with an integrated anion receptor site capable of accommodating chiral carboxylate and phosphate anions, **38**, was used to assess this possibility.^26b^ Ligand **38** has time-averaged *C*_*s*_ symmetry. Variable-temperature NMR spectroscopy showed that the barrier to inversion of the biphenyl groups is ≤10 kcal/mol. The ligand can adopt four different conformations. Two are *C*_2_-symmetric (*R*_*a*_,*R*_*a*_ and *S*_*a*_,*S*_*a*_) and thus chiral, and in the absence of chiral group, they are necessarily present in equal amounts. As a result of the presence of a pseudochiral center, two *C*_*s*_-symmetric and evidently achiral structures are possible (*R*_*a*_,*r*,*S*_*a*_ and *R*_*a*_,*s*,*S*_*a*_). Symmetrically substituted *E*- and *Z*-olefins can coordinate to each structure in two different ways; thus, for each olefin eight different complexes are possible. Four out of the eight complexes in each group can interconvert via tropoisomerization, whereas interconversion of the remaining isomers requires decoordination–recoordination of the olefin.

In the presence of chiral anions, the mirror symmetry of the ligand is lost, resulting in separate ^31^P NMR signals. No further split of the signals was observed at low temperature in the presence of BINOL phosphate, indicating the presence of a single conformer.

Coordination of the model *E*-olefin fumaronitrile to the ligand in the presence of acetate and no chiral anion gave rise to a single diastereomer, evidently present as a racemate, whereas in the presence of dimethyl fumarate, one major and one minor diastereomer that were in rapid equilibrium at room temperature were obtained, as evidenced by ^31^P NMR spectroscopy. In the presence of chiral anions such as carboxylates and phosphates, stable, non-interconverting diastereomeric complexes were formed; with (*S*)-2-hydroxy-3-methylbutyrate the homochiral diastereomers were formed in a ca. 1.5:1 ratio. There was slow exchange between free and bound chiral anion at low temperature. In contrast, a single complex was obtained with BINOL-phosphate bound to the anionic receptor site. With a *cis*-olefin, diethyl maleate, mixtures of diastereomers were obtained with the two chiral anions.

In catalytic reactions, the chiral transfer relies solely on the long-distance interaction between the tropos units and the chiral anion. Therefore, the observation of moderate enantioselectivites in the catalytic reactions was not surprising. In the presence of (*S*)-2-hydroxy-3-methylbutyrate, for example, Pd-catalyzed allylic alkylation of *rac*-1,3-diphenylpropenyl carbonate (carbonate was used instead of acetate to avoid replacement of the chiral carboxylate by acetate) with malonate gave the product with 57% ee under the optimized conditions. With benzylamine as the nucleophile, the product was obtained with 66% ee.

The analogous reaction using *rac*-3-cyclohexenyl carbonate in the presence of the same chiral carboxylate anion resulted in racemic product. In contrast, in the presence of BINOL phosphates, the product was obtained with up to 43% ee, demonstrating that the structure of the catalytic site can be fine-tuned by the choice of chiral anion.

## The Clue to the Wide Scope of the Flexible PHOX

Our two groups decided to examine the reason for the exceptionally wide substrate tolerance of ligand **17** (see Scheme 4), which in contrast to the “parent” PHOX ligand gives products with high enantiomeric purity from a wide range of “broad” as well as “narrow” substrates in Pd-catalyzed allylic alkylations (Scheme 4).^3^

**Scheme 10:**
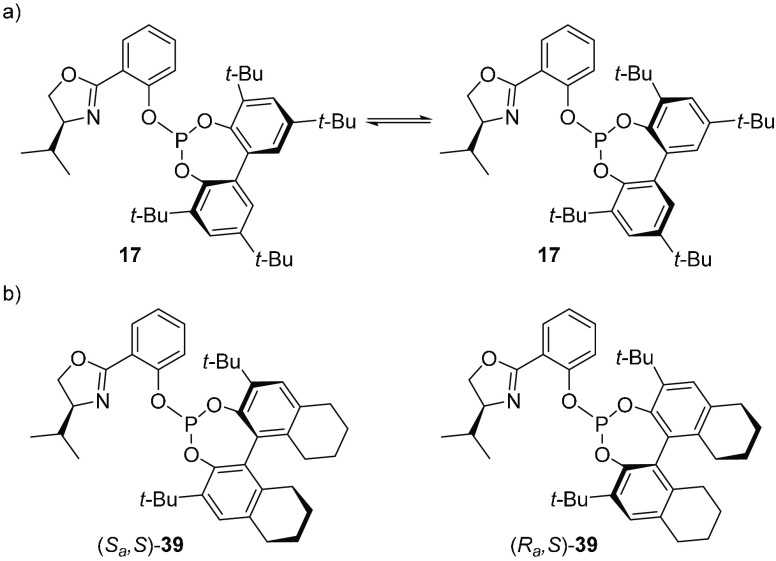
(a) Tropoisomerization of Ligand **17**; (b) Conformationally Rigid Phosphite–Oxazoline Ligands (*S*_a_,*S*)- and (*R*_a_,*S*)-**39**, Analogues of **17**

At room temperature, the ligand gave rise to a single ^31^P NMR signal, which gradually broadened and split into two signals at around −20 °C (Scheme 10a). In order to gain insight into the conformational preferences of the ligand for different types of substrates, catalytic reactions with the analogous rigid, atropos, ligands (*S*_a_,*S*)- and (*R*_a_,*S*)-**39** were studied (Scheme 10b).

Ligands (*S*_a_,*S*)- and (*R*_a_,*S*)-**39** provided largely different results (Table 1). With the model substrate *rac*-1,3-diphenylpropenyl acetate, high enantioselectivities (>99% ee) and high reactivities were observed with (*S*_a_,*S*)-**39** as well as with flexible ligand **17**, whereas the use of (*R*_a_,*S*)-**39** resulted in a product with the opposite absolute configuration with low enantioselectivity (20% ee). A 1:1 mixture of the two atropos ligands gave the product with 90% ee, demonstrating a major difference in reactivity between the catalysts with the two ligands. The results show that the absolute configuration of the product was determined by the biaryl part of the molecule.

**Table 1:**
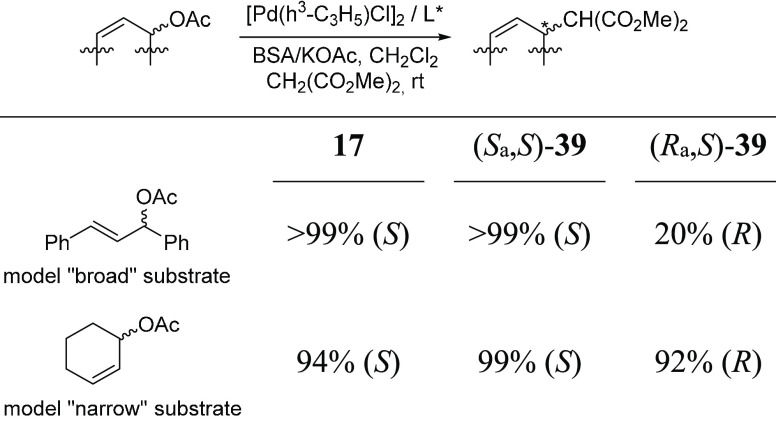
Pd-Catalyzed Allylic Alkylation of Model “Broad” and “Narrow” Substrates with Ligands **17**, (*S*_a_,*S*)-**39**, and (*R*_a_,*S*)-**39**^a^

a Full conversion was attained in all cases.

**Figure 17:**
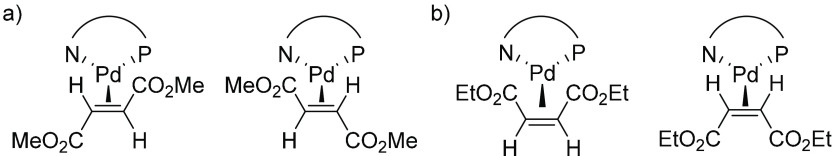
Representations of the two possible Pd(0) complexes that can be formed with (a) *E*- and (b) *Z*-olefins.

The assumption of a late transition state motivated studies of model olefin complexes, again with dimethyl fumarate and diethyl maleate as mimics of the transition states.^56^ Because of their symmetry, each olefin can coordinate with either of their two faces, and because of their symmetry, each olefin gives rise to two possible complexes from each ligand (Figure 17). Comparison of the NMR spectra of the olefin complexes with the flexible ligand, which were unaffected by cooling, with those of the complexes with rigid ligands (*S*_a_,*S*)- and (*R*_a_,*S*)-**39** revealed, to our surprise, that the flexible ligand adopted the (*S*_a_,*S*) configuration with both types of olefins. Even more surprising was the fact that the olefins coordinated with the same face in the diastereomeric rigid ligands in spite of the observation of products with different absolute configurations from the ligands.

**Figure 18:**
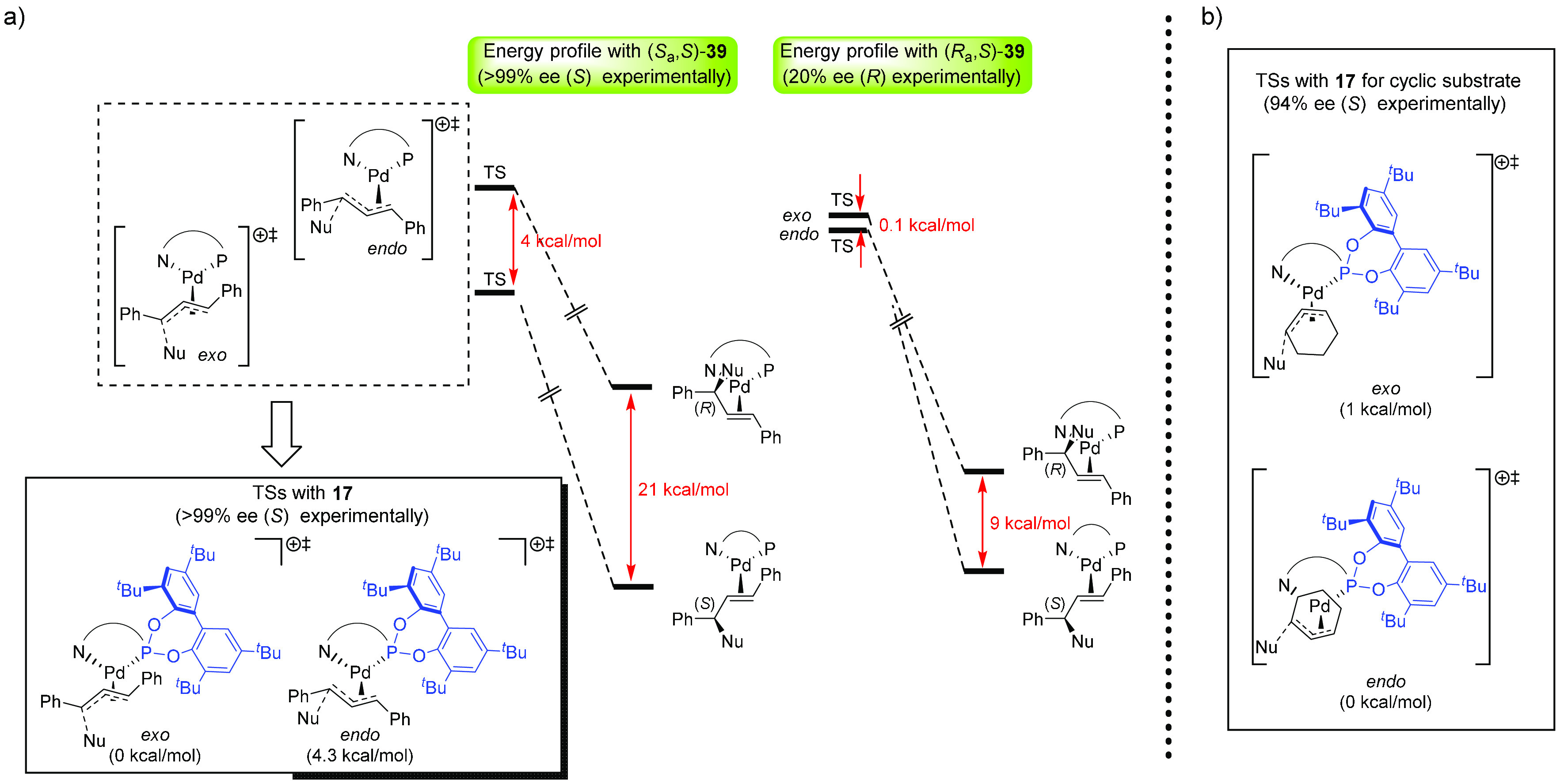
(a) Calculated relative energies of the product Pd–olefin complexes, the transition states for nucleophilic attack at the Pd−η^3^-allyl complexes for the “broad” substrate with (*S*_a_,*S*)-**39** and (*R*_a_,*S*)-**39**, and the transition states for the “broad” substrate with ligand **17**. (b) Calculated relative energies of the transition states for the “narrow” substrate with ligand **17**.

DFT calculations by P. O. Norrby on the olefin complexes with the “authentic” substrates confirmed the conclusions from the model olefin complexes (Figure 18). DFT transition state calculations, with ammonia as a nucleophile approaching the allyl group from the side trans to P,^47^ showed that the lowest-energy transition states for the reactions of *rac*-1,3-diphenylpropenyl acetate and *rac*-3-cyclohexenyl acetate led to the products observed and predicted from studies of olefin complexes when ligands **17** and (*S*_a_,*S*)-**39** were used. In contrast, the lowest-energy transition states in reactions using ligand (*R*_a_,*S*)-**39** did not lead to the most stable olefin complexes, thus explaining why the product does not correspond to the model olefin complex (Figure 18).

The flexible ligand thus adopts the (*S*_a_,*S*) configuration in reactions with both “broad” and “narrow” substrates. The explanation for the exceptionally wide substrate scope must thus be found in the ability of the ligand to adjust the size of the substrate-binding pocket to the steric requirements of the substrate and not in a change of configuration. The explanation for the excellent performance of ligand **17** also in a range of other asymmetric catalytic reactions is most probably also to be found in its ability to adapt to the demands of each particular substrate. The results underline the importance of adaptation in synthetic catalytic systems.

## Conclusion

Conformationally flexible ligands with the ability to undergo stereomutation and thereby change their size and form in order to optimize non-covalent interactions with the substrate have proven to often outperform their rigid counterparts in asymmetric metal-catalyzed reactions.

In this Account, we have presented experimental and theoretical studies performed in our two groups aimed at gaining insight into the conformational preferences of such ligands and the consequences of their preferred conformations in asymmetric catalysis. Whereas some tropos ligands adopt different configurations with different substrates, a PHOX analogue with the diphenylphosphine group replaced by a tropos unit does not change configuration but is able to adapt the binding site to specific reactions and substrates, resulting in exceptionally wide substrate scope and versatility in a number of catalytic processes such as catalytic hydrogenations, hydroborations, and intermolecular Heck reactions.

It is our hope that this Account will inspire the design and use of new of flexible ligands based on biaryls or other flexible motifs.
